# Activation of C6 glioblastoma cell ceruloplasmin expression by neighboring human brain endothelia-derived interleukins in an in vitro blood–brain barrier model system

**DOI:** 10.1186/s12964-014-0065-7

**Published:** 2014-10-14

**Authors:** Ryan C McCarthy, Daniel J Kosman

**Affiliations:** Department of Biochemistry, University at Buffalo, School of Medicine and Biomedical Sciences Buffalo, Farber Hall Room 140, 3435 Main St., Building 26, Buffalo, NY 14214-3000 USA

**Keywords:** IL-1β, IL-6, Ceruloplasmin, Glial cells, Brain microvascular endothelial cells, Blood–brain barrier, Iron, Cytokines, Inflammation

## Abstract

**Background:**

Iron transport across the blood–brain barrier (BBB) involves the cooperation of brain microvascular endothelial cells (BMVEC) and their neighboring astrocytes. Astrocytes secrete a soluble form of ceruloplasmin (sCp) which, in turn, acts to export iron from ferroportin (Fpn) on the basolateral surface of BMVEC. Although regulation of astrocyte sCp gene expression has been demonstrated to be influenced by interleukin-1 beta (IL-1β) and interleukin-6 (IL-6), the role of neighboring BMVEC in this regulation has yet to be determined and is the basis for this work.

**Results:**

We provide evidence that human BMVEC (hBMVEC) IL-1β and IL-6 positively influence the expression of sCp transcript by neighboring C6 glioma cells (astrocytes). The effect of hBMVEC on C6 glioma sCp expression at the level of transcript and protein was repressed via the addition of IL-1β and IL-6 pathway inhibitors (IL-1 receptor antagonist protein and SC144, respectively). Stimulation of hBMVEC interleukin gene expression by apical exposure to bacterial endotoxin lipopolysaccharide significantly enhanced hBMVEC-mediated C6 glioma sCp gene expression.

**Conclusion:**

hBMVEC influence the gene expression of neighboring C6 glioma sCp. This change in gene expression is mediated by the secretion of IL-1β and IL-6 from hBMVEC. Furthermore, the hBMVEC-induced increase in neighboring C6 glioma sCp gene expression leads to an increased rate of hBMVEC iron efflux. Taken together, our results indicate that hBMVEC-secreted cytokine activity increases the gene expression of neighboring C6 glioma sCp, which reciprocally acts on basolateral hBMVEC Fpn to enhance brain iron import.

**Electronic supplementary material:**

The online version of this article (doi:10.1186/s12964-014-0065-7) contains supplementary material, which is available to authorized users.

## Introduction

Iron, a first-row transition metal, is essential for cellular respiration [1,2]. As the major user of metabolic energy (on a per-weight basis) the central nervous system (CNS) strongly relies on iron while at the same time is highly vulnerable to iron-induced oxidative stress. Indeed, progressive accumulation of iron in a normal aging brain [3] or pathologic alterations of its homeostasis can be the cause of or contribute to the cellular degeneration observed in many neurologic disorders [3–6]. The export of iron from the basolateral surface of the brain microvascular endothelial cells (BMVEC) of the blood–brain barrier (BBB) is mediated by several different layers of regulation by neighboring astrocytes [7]. In a model BBB system, C6 glioblastoma cells (astrocytes) inhibit the export of iron from BMVEC via secretion of the peptide hormone hepcidin which when bound to its receptor ferroportin (Fpn), induces its internalization and degradation [7–9]. Alternatively, C6 cells increase the export of iron from the basolateral surface of BMVEC (and thus import into brain) via the secretion of the soluble multi-copper oxidase ceruloplasmin (sCp) [7,10].

C6 glioma Cp expression can be modulated by interleukin-1β (IL-1β) [11,12]. IL-1β binding to its receptor, IL-1 receptor 1 (IL-1R1), modulates the activity of mitogen-activated protein kinases (MAPK) in mammalian cells [13]; this activity has been demonstrated in C6 cells in culture [11]. Furthermore, IL-1β has been shown to increase production of C6 glioma-secreted sCp protein [14]. IL-1β is produced by monocytes, macrophages, fibroblasts, epidermal cells, and also by endothelial cells [13,15–17].

Alternatively, cellular Cp gene expression can be modulated by IL-6 [18,19]. Signal transduction by IL-6 requires the binding to its receptor, the IL-6 receptor (IL-6R) on the cell surface. This binding event leads to association of the receptor with the signal transducer gp130 which initiates a downstream signaling cascade leading to the modulation of gene expression [20]. Reports from the literature suggest that human endothelial cells express and secrete IL-6 [21,22].

A direct link between IL-1β or IL-6 secretion from BMVEC and the downstream effect of increasing astrocyte sCp gene expression has not been established; this link is likely part of the cell communication and signaling network that results in iron flux across the BBB. Here, we investigate this potential link in the context of brain iron influx. We use a human BMVEC line (hBMVEC) to demonstrate that hBMVEC increase the gene expression of C6 glioma sCp. Furthermore, we confirm that hBMVEC express and secrete IL-1β and IL-6 into their extracellular space. We show that the increase in C6 glioma sCp gene expression in the presence of hBMVEC can be repressed through the inhibition of either the IL-1R1 or the gp130 signal transducer molecule involved in IL-6 modulated gene activation. Finally, we demonstrate that hBMVEC cytokine release, and thus stimulation of astrocyte sCp gene expression, can be enhanced by apical exposure of hBMVEC to bacterial endotoxin lipopolysaccharide. These data are the first to define a likely cell communication and signaling network between BMVEC and astrocytes at the BBB that enhances iron transport across the BBB and into the brain.

## Results

### hBMVEC increase C6 glioma ceruloplasmin mRNA abundance

**Figure 1:**
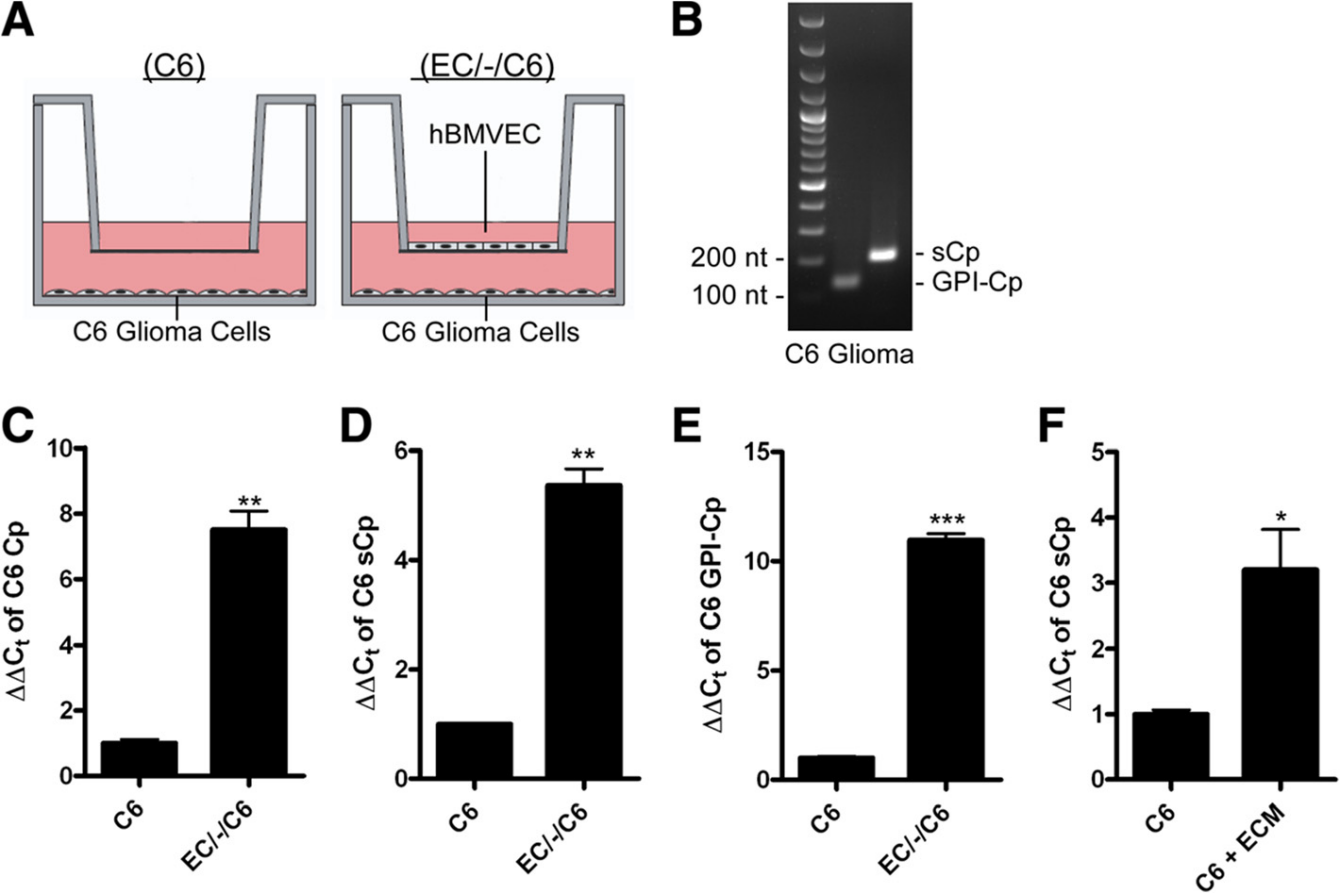
**hBMVEC up-regulate C6 glioma sCp gene expression. (A)** Illustration of C6 glioma cells seeded alone (C6 only) or distal to hBMVEC (EC/-/C6) in transwell. **(B)** RT-PCR of total C6 glioma RNA examining the presence of sCp and GPI-Cp transcript. **(C-E)** qPCR of isoform non-specific Cp **(C)**, sCp **(D)**, or GPI-Cp **(E)** transcript from total RNA collected from C6 glioma cells grown alone (C6) or distal to hBMVEC (EC/-/C6). **(F)** Serum-free basal media was collected from hBMVEC grown alone in transwell (ECM) and was applied to C6 glioma cells for 24 h. Data represents relative gene expression of sCp in C6 glioma cells obtained by qPCR in control or with ECM addition. A series of paired t-tests were used to determine the significance of the data. *P < 0.05, **P < 0.01, ***P < 0.001. Data are represented as means ± s.d. (n = 3, technical replicates).

We have demonstrated previously that C6 glioma cells grown distal to hBMVEC in a transwell culture system (top well: hBMVEC; bottom well, bottom surface: C6 cells) significantly enhance hBMVEC iron efflux into the basal chamber via secretion of C6 cell sCp [7]. Also observed, was an increase in C6 cell sCp transcript mediated by neighboring hBMVEC [7]. We took advantage of this transwell model system to determine the mechanism by which hBMVEC increase C6 cell sCp gene expression in co-culture. Two distinct cellular orientations were used for our experiments which included C6 glioma cells seeded either alone (C6 only) or spatially adjacent but distal to hBMVEC (EC/-/C6) (Figure 1A).

After the cells were grown to confluency in their respective orientations, the media in the basal (bottom) chamber was switched to RPMI 1640 minus serum to mimic the interstitial fluid of the brain. Cells were incubated with this new media for an additional 24 h to be consistent with previous work [7]. Total RNA was extracted from the hBMVEC and the C6 glioma cells under the different orientations as indicated. Transcripts for both splice variants of Cp (sCp and GPI-Cp) were identified in C6 glioma cells (Figure 1B) and their relative abundance under each orientation was assessed quantitatively using qPCR. We observed a 7-fold increase in the transcript abundance of splice variant non-specific Cp abundance in C6 glioma cells seeded spatially adjacent to hBMVEC compared to their respective C6 only control (Figure 1C). The increases in sCp and GPI-Cp mRNA were 5- and 10-fold (Figures 1D and 1E). The effect on C6 glioma sCp transcript expression was not influenced by serum from the apical chamber (Additional file 1: Figure S1). Similarly, there was only a small increase in the abundance of sCp transcript in hBMVEC grown distally to C6 cells (Additional file 1: Figure S2). Note that we do not make comparisons between varying transcript targets (sCp vs. GPI-Cp) or between hBMVEC and C6 glioma sCp message as this is not possible with qPCR methodology. Previous data has shown there is no significant difference in sCp message in C6 cells grown either in the distal (EC/-/C6) and proximal (EC/C6) orientations [7].

We tested if the increase in sCp transcript abundance within C6 glioma cells required co-culture with hBMVEC or whether this increase was due to a stable hBMVEC exocrine factor retained in hBMVEC conditioned media. To test the latter model, hBMVEC were seeded alone in transwell and grown to confluence. Their basal media was exchanged with serum-free media to mimic the brain interstitial fluid, and the hBMVEC were incubated for 24 h to “condition” the basolateral media. This basolateral hBMVEC-conditioned media (ECM) was then applied to C6 glioma cells grown in 66-mm dishes and allowed to incubate for 24 h. Total RNA was isolated from the C6 glioma cells and qPCR revealed a significant, 3-fold increase in C6 glioma sCp message (Figure 1F). These data suggest the secretion of the putative hBMVEC factor into the basal chamber that stimulated C6 glioma sCp gene expression did not require but was enhanced by signaling from C6 glioma cells to hBMVEC.

### hBMVEC express cytokines IL-1β and IL-6 which are secreted from their basolateral surface

**Figure 2:**
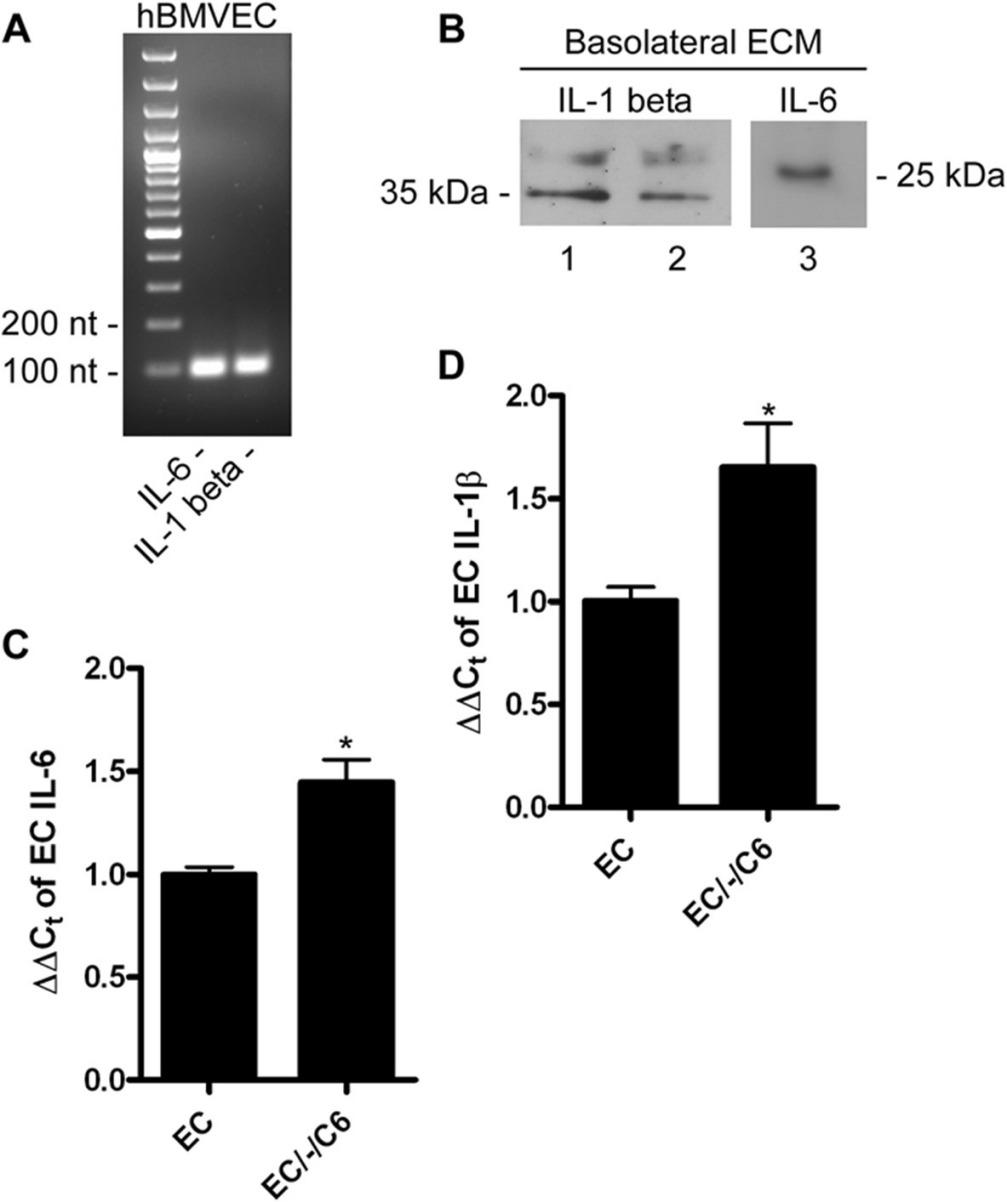
**Interleukin-1β and interleukin-6 are expressed by hBMVEC. (A)** RT-PCR of total RNA from hBMVEC examining the presence of IL-6 and IL-1β transcript. **(B)** Immunoblot of basal hBMVEC-conditioned media (Basal ECM) probing for IL-1β (lanes 1 (20 μL sample loaded) and 2 (10 μL sample loaded)) and IL-6 (lane 3). **(C-D)** qPCR of hBMVEC IL-6 **(C)** or IL-1β **(D)** transcripts from total RNA isolated from hBMVEC grown either alone (EC) or distal to C6 glioma cells (EC/-/C6). A series of paired t-tests were used to determine the significance of the data. *P < 0.05. Data are represented as means ± s.d. (n = 3, technical replicates).

Data indicate that Cp transcript abundance in astrocytes can be increased through exposure to the cytokines IL-1β or IL-6 [11,12,14,18,19] suggesting the model that either or both of these cytokines were the factor(s) secreted by hBMVEC into the basal chamber. Thus, total RNA was isolated from hBMVEC; RT-PCR analysis revealed the presence of transcripts for both factors (Figure 2A). We demonstrated also the presence of the IL-1β and IL-6 protein in basolateral hBMVEC-conditioned media. To do this hBMVEC were grown alone in transwell. The basolateral media was switched to media without serum and the cells were incubated for an additional 24 h to condition the media as above. Western blot analysis of this hBMVEC-conditioned media (ECM) demonstrated the presence both IL-1β and IL-6 protein, confirming that hBMVEC secrete both cytokines from their basal surface into the extracellular space (Figure 2B). Of note is that the abundance of hBMVEC IL-6 and IL-1β transcripts were increased by co-culture in transwell with C6 glioma cells (Figures 2C and 2D, respectively). This effect correlates with the 7-fold increase C6 glioma sCp transcript when grown in transwell together with hBMVEC (Figure 1D) in comparison to the 3-fold increase in C6 cells incubated with basal ECM (not in transwell; Figure 1F).

### IL-1β and IL-6 act additively to increase C6 glioma sCp mRNA and protein

**Figure 3:**
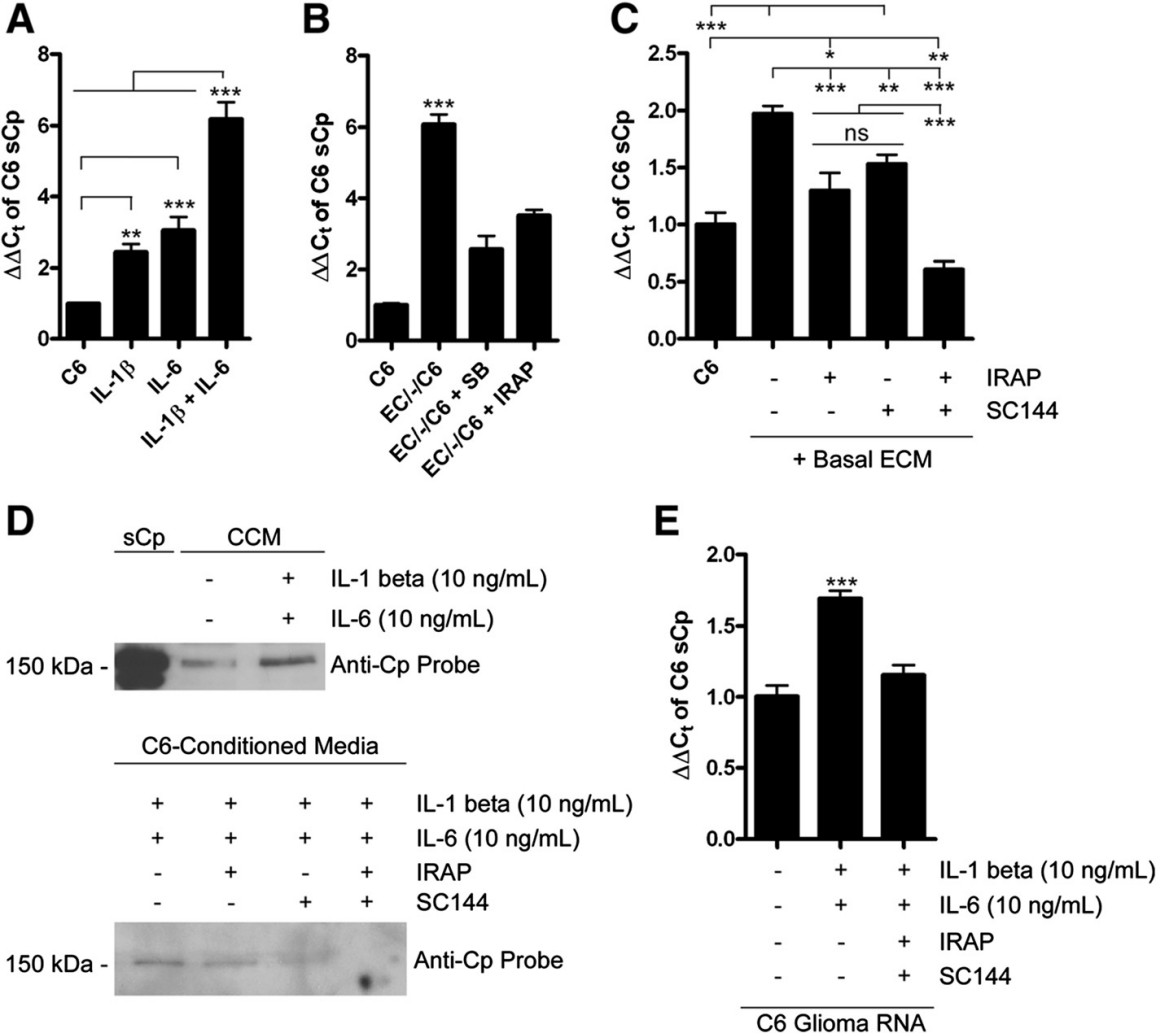
**hBMVEC IL-6 and IL-1β enhance C6 glioma sCp gene expression. (A)** qPCR of C6 glioma sCp transcript from total RNA isolated from C6 cells incubated with either nothing, IL-1β (10 ng/mL), IL-6 (10 ng/mL), or both for 24 h. **(B)** SB203580 and IRAP, which inhibit the action of hBMVEC-secreted IL-1β, repress the ability of hBMVEC-secreted IL-1β to enhance C6 glioma sCp gene expression. Relative C6 glioma sCp transcript abundance grown either alone (C6), or distal to hBMVEC (EC/-/C6) with or without 20 μM SB203580 or 1 μg/mL IRAP for 24 h. **(C)** Relative abundance of C6 sCp from total RNA of C6 glioma cells seeded alone with or without the addition of basal hBMVEC-conditioned media (Basal ECM) and the IL-1β inhibitor IRAP (1 μg/mL) or the IL-6 inhibitor SC144 (20 μM). Drugs were added to C6 glioma cells for 1 h prior to the addition of Basal ECM for an additional 1 h before total RNA was isolated. **(D)** Immunoblot probing for Cp in C6-conditioned media in which the cells had been incubated with either nothing, IRAP (1 μg/mL), SC144 (20 μM), or both for 1 h prior to the addition of both IL-1β (10 ng/mL) and IL-6 (10 ng/mL), or nothing for an additional 6 h. Purified human sCp (20 ng) is used as a positive control in the top blot. **(E)** Total RNA isolated from C6 glioma cells treated as described in **(D)** were analyzed for sCp transcript abundance by qPCR. One-way ANOVA analyses of the variance parameters were used to determine the significance of the data. *P < 0.05, **P < 0.01, ***P < 0.001, ns is not significant. Data are represented as means ± s.d. (n = 3, technical replicates).

Since both IL-1β and IL-6 are secreted from the basolateral surface of hBMVEC, we examined the direct effect of added recombinant cytokine on C6 glioma sCp abundance. C6 glioma cells were incubated for 6 h in the absence of either cytokine (control) and in the presence of IL-1β, IL-6, or both. Total RNA was isolated from the C6 cells and analyzed for sCp transcript abundance by qPCR. The cytokines IL-1β (10 ng/mL) and IL-6 (10 ng/mL) had an additive effect with respect to the increase of C6 cell sCp transcript abundance (Figure 3A). These data are fully consistent with literature data that show IL-6 and IL-1β mediate increases in sCp gene expression [11,12,18,19].

### SB203580 and interleukin-1 receptor antagonist protein (IRAP) repress the induction of C6 glioma sCp gene expression by hBMVEC

Although we have demonstrated that IL-1β is expressed and secreted by hBMVEC, and confirmed in our system that addition of this cytokine to C6 cells induced sCp expression, these two findings do not specifically show that in the hBMVEC/C6 cell co-culture, the increase in C6 cell sCp was due to hBMVEC IL-1β. Thus we examined if pharmacological inactivation of the IL-1 receptor (IL-1R) or the downstream MAPK pathway in C6 cells would repress the effect of hBMVEC on sCp expression. Interleukin-1 receptor antagonist protein (IRAP) inhibits IL-1R activation by IL-1β [13,23] and IRAP has been effectively used in C6 cells to inhibit sCp gene expression induced by IL-1β [24]. Furthermore, in binding its receptor, IL-1R, the IL-1β activates three independent MAPK pathways, one of which involves the p38 MAP kinase [13]; SB203580 is a specific inhibitor of p38 MAP kinase activated by IL-1β [11,25]. Addition of SB203580 to C6 cells has been documented to repress the IL-1β-induced increase in Cp transcript in those cells [11]. Thus, either 1 μg/mL IRAP or 20 μM SB203580 were added to separate basolateral chambers of transwells with hBMVEC grown distal to C6 glioma cells (EC/-/C6). After a 24 h incubation period, total RNA was isolated from the C6 glioma cells and qPCR was performed. The addition of either IRAP or SB203580 to the basolateral chamber partially repressed the hBMVEC-induced increase in C6 cell sCp message (Figure 3B). These data are consistent with the model that IL-1β secreted from hBMVEC enhances C6 sCp gene expression at least in part by activating the IL-1R-dependent signaling pathway.

### The hBMVEC-mediated induction of C6 glioma sCp gene expression is repressed by pharmacological agents that block the activation of IL-1β and IL-6 receptors

Blocking the IL-1R pathway only partially repressed the activation of sCp expression in hBMVEC co-cultures. Thus, we hypothesized IL-6, secreted from hBMVEC, also contributed to this activation. Promotion of Cp gene expression by IL-6 has been observed previously [18,19]. Induction of gene expression by IL-6 begins with the cytokine binding to the IL-6R. This ligand binding event induces the association of gp130 which is required for signal transduction and downstream gene activation [20]. Phosphorylation of serine residue 782 on gp130 down-regulates gp130 glycosylation and leads to suppression of its downstream signaling pathway [20]. SC144, a recently identified inhibitor of gp130, was described to induce this phosphorylation of gp130 Ser782 and thus inhibit gp130 signal transduction [20]. We used SC144 examine whether hBMVEC-secreted IL-6 contributed to C6 glioma sCp gene expression.

C6 glioma cells were grown in 6-well tissue culture dishes to confluence. The media was exchanged with serum-free media plus or minus either IRAP (1 μg/mL), SC144 (20 μM), or both and the cultures were allowed to incubate for 1 h. The media was removed and replaced by hBMVEC-conditioned media as above (cells grown alone) and the C6 glioma cells were incubated for an additional 1 h, maintaining the initial concentration(s) of the two drugs. The incubation period was shortened in comparison to Figures 3A and 3B due to SC144 cytotoxicity at 24 h. Total RNA was isolated from the C6 glioma cells and qPCR was performed to determine the transcript abundance of sCp in each condition. In the 1-h incubation period, hBMVEC basal conditioned media (Basal ECM) caused a 2-fold induction in C6 glioma sCp gene expression (Figure 3C). This induction was repressed by both IRAP and SC144 but was completely inhibited with the addition of both drugs together (Figure 3C).

Since IRAP and SC144 treatment diminished the hBMVEC-induced increase in C6 glioma sCp transcript expression, we predicted that this observation would translate to the level of protein expression. First, we confirmed by western blot that IL-1β and IL-6 incubated with C6 glioma cells for 6 h yielded an increase in the secretion of sCp from C6 glioma cells into the media (Figure 3D). Purified IL-6 and IL-1β were used here instead of hBMVEC in transwell or hBMVEC-conditioned media because hBMVEC express endogenous sCp at low-levels [26] thus contributing the sCp detected in the C6 cell-conditioned medium. C6 glioma cells were then pre-treated with or without IRAP, SC144, or both for 1 h, followed by incubation with cytokines for an additional 6 h. After this induction period, the media was analyzed for the relative abundance of sCp by immunobloting. We found that IRAP, and to a greater extent SC144 reduced the amount of sCp present in the C6 glioma-conditioned media (Figure 3D); furthermore, no sCp was detected in the media when IRAP and SC144 were added together (Figure 3D). Analysis of the total RNA collected from C6 glioma cell samples processed in parallel with those used for the western blot data as in Figure 3D demonstrated a corresponding decrease in sCp transcript abundance (Figure 3E).

### Induction of C6 glioma sCp gene expression by hBMVEC leads to an increase in the rate of hBMVEC ^59^Fe-efflux

**Figure 4:**
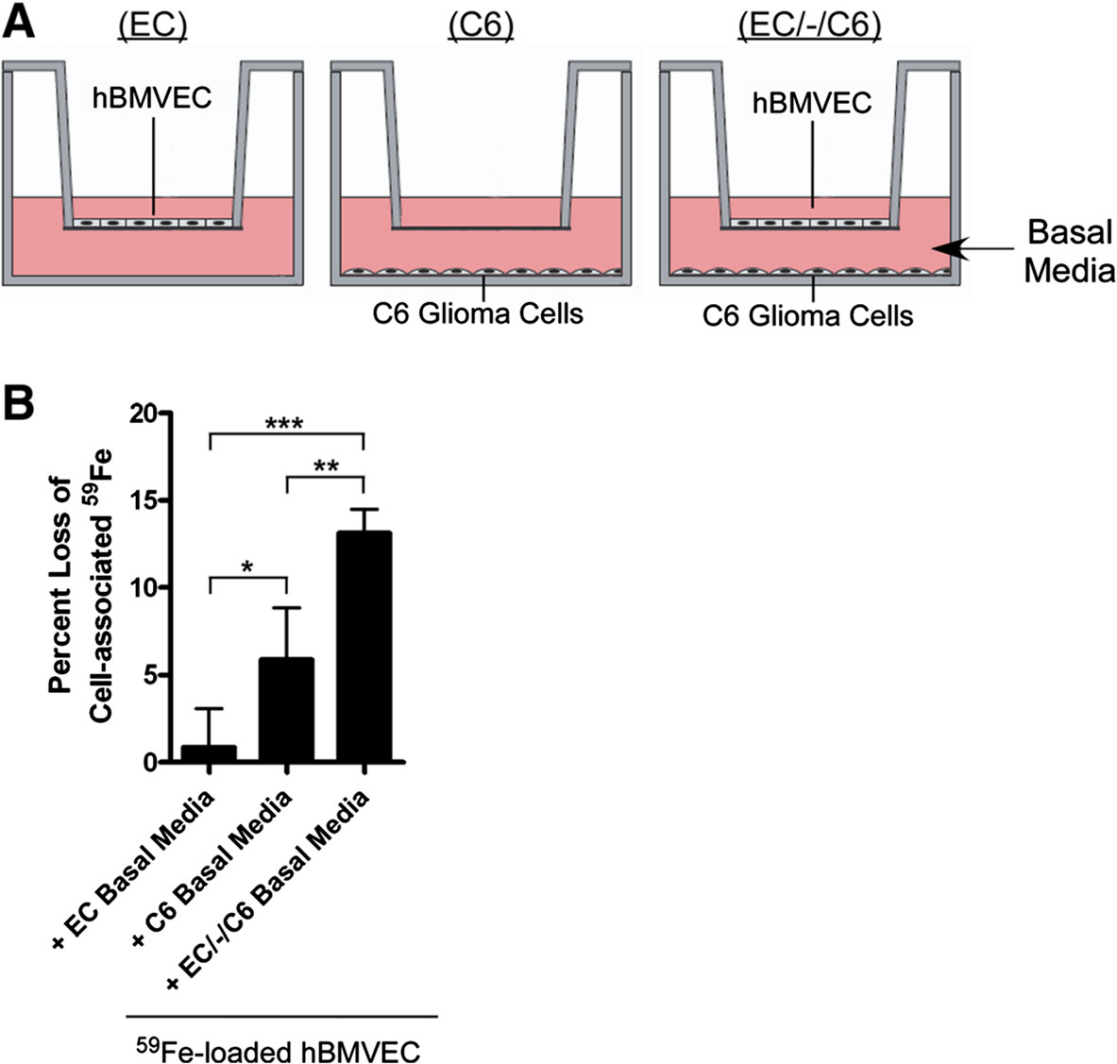
**Increased C6 glioma sCp expression enhances hBMVEC** ^**59**^ **Fe-efflux. (A)** Illustration of *in vitro* co-culture setup in transwells. In all cases the apical chamber contained RPMI1640 plus serum while the basolateral chamber was devoid of serum. **(B)** hBMVEC were loaded for 24 h with ^59^Fe^II^-citrate plus ascorbate and then iron efflux assays were performed. The percent loss of cell-associated ^59^Fe from hBMVEC after 24 h relative to t = 0 h was monitored. Efflux buffer was basolateral media conditioned for 24 h with the cellular orientations depicted in A. Significance of the data was determined using one-way ANOVA statistical analysis of the variance parameters. *P < 0.05, **P < 0.01, ***P < 0.001. Data are represented as means ± s.d. (n = 8, experimental replicates).

Our data indicate that hBMVEC-secreted cytokines IL-1β and IL-6 activate sCp gene expression in C6 glioma cells. We have previously demonstrated that C6 glioma-secreted sCp enhances basolateral hBMVEC ^59^Fe efflux from Fpn [7]; immunodepletion experiments were used to confirm that sCp was indeed the species in the C6 secretome that supported the increase in hBMVEC iron mobilization [7]. Therefore, we tested the hypothesis that cytokine-dependent increased C6 glioma sCp expression would lead to an increase in iron efflux activity from hBMVEC Fpn. Thus, we prepared transwells with hBMVEC alone (EC Only), C6 glioma cells alone (C6 Only), or both cells seeded distal to one another (EC/-/C6) (Figure 4A). After the cells had grown to confluence the media in the apical chamber was replaced with fresh RPMI1640 with serum while the basal chamber media was replaced with serum-free RPMI1640. Transwells were incubated for 24 h to allow the basal chamber media to become “conditioned”. After 24 h, the basal chamber media was collected and used as efflux media for hBMVEC monolayers that had been loaded for 24 h with ^59^Fe^II^-citrate plus Asc in RPMI1640 without serum. After a 24 h efflux period, we observed a significant enhancement of hBMVEC ^59^Fe-efflux when the efflux media was conditioned with EC/-/C6 as compared to medium that was conditioned by either cell type alone (Figure 4B). These data are consistent with the model that hBMVEC-induced up-regulation of C6 glioma sCp gene expression has a functional role in stimulating Fpn-dependent iron efflux from hBMVEC. The hBMVEC cytokine-mediated increase in glial cell sCp described herein is not functionally adequate to override the effect of glial cell secreted hepcidin on hBMVEC iron efflux when hBMVEC are seeded proximal to C6 glioma cells in a model BBB system [7].

### Lipopolysaccharide positively influences the hBMVEC-induced increase in C6 glioma sCp transcript abundance

**Figure 5:**
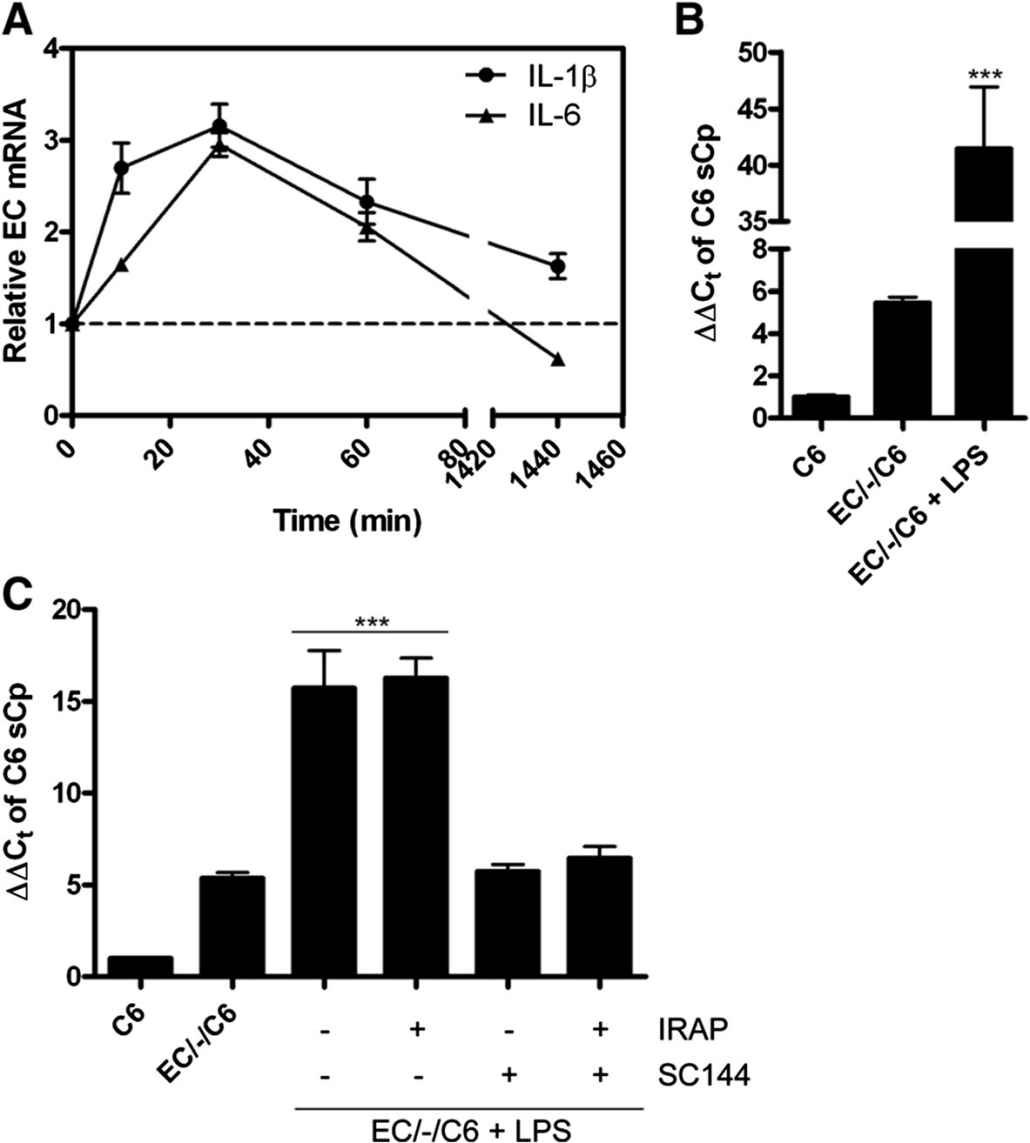
**LPS-induced activation of hBMVEC basolateral iron efflux through modulation of hBMVEC interleukins with subsequent C6 glioma cell sCp gene activation. (A)** LPS (100 μg/mL) was added to hBMVEC for the times indicated before total hBMVEC RNA was collected and assayed for IL-1β and IL-6 transcript abundance via qPCR. **(B)** Total RNA was isolated from C6 glioma cells seeded distal to hBMVEC incubated with or without the addition of LPS (100 μg/mL) to the apical chamber for 24 h. Soluble Cp transcript abundance was assessed via qPCR. **(C)** C6 glioma cells were incubated alone or distal to hBMVEC for 20 h prior to the addition of LPS (100 μg/mL) to the apical chamber and IRAP (1 μg/mL) and/or SC144 (20 μM) to the basal chamber for an additional 4 h as indicated. Total C6 glioma RNA was isolated and assayed for sCp transcript abundance via qPCR. Significance of the data was determined using either a paired t-test or one-way ANOVA statistical analyses. ***P < 0.001. Data are represented as means ± s.d. (n = 3, technical replicates).

Lipopolysaccharide (LPS) is a bacterial endotoxin used experimentally as a positive modulator of inflammation (i.e. interleukin up-regulation) in mammalian cell culture [27,28]. An increase in the polarized release of cytokines (most notably IL-6) from BMVEC via the addition of 100 μg/mL LPS has been reported [28]. We tested the hypothesis that addition of LPS at this concentration to hBMVEC would further increase C6 glioma sCp transcript abundance via increased cytokine secretion from hBMVEC. Indeed, an increase in the transcript abundance of IL-6 and IL-1β in hBMVEC treated for short time periods with LPS (100 μg/mL) was noted (Figure 5A). LPS addition (100 μg/mL) to the apical chamber of hBMVEC grown distal to C6 glioma cells induced a 40-fold increase in sCp transcript abundance in the C6 glioma cells over 24 h (Figure 5B). To demonstrate that the effect of LPS on C6 glioma cell sCp expression was dependent upon hBMVEC, we examined the effect of LPS addition to the apical chamber of transwells in which C6 glioma cells were grown alone in the bottom chamber; in this experiment, LPS had no positive effect on C6 cell sCp transcript abundance (Additional file 1: Figure S3).

We hypothesized that hBMVEC IL-1β and/or IL-6 were likely responsible for the increase in C6 glioma sCp gene expression when hBMVEC were stimulated apically with LPS as in Figure 5B. To test this, we grew C6 glioma cells either alone (C6) or distal to hBMVEC in transwell (EC/-/C6). Fresh RPMI 1640 with serum was added to the apical chamber and fresh RPMI 1640 without serum was added to the basal chamber 24 h prior to the collection of C6 glioma RNA. Twenty hours after the media was exchanged, LPS (100 μg/mL) was added to the apical chamber and the inhibitors IRAP (1 μg/mL) and SC144 (20 μM) were added to the basal chamber as indicated. The cells were incubated for an additional 4 h before total RNA was isolated from the C6 glioma cells and analyzed for the relative transcript abundance of sCp by qPCR (Figure 5C). As shown, the IL-6 inhibitor SC144 blocked the action of LPS-induced hBMVEC on C6 glioma cell sCp gene expression, while the IL-1β inhibitor IRAP had no notable effect (Figure 5C). Note that complete inhibition of the LPS effect is represented by the level of sCp transcript abundance in the EC/-/C6 without LPS condition (i.e. approx. 5) and not by the level of sCp transcript abundance in the C6 alone condition (i.e. approx. 1). The absence of an IRAP effect (that is, a down-regulation of the IL-1 β pathway) in this instance could be the result of a dominant IL-6 dependent activation of sCp transcript production.

## Discussion

In the brain parenchyma, a tissue closed off from the peripheral circulation, cell-to-cell communication is imperative for the transport of essential metabolites. Without paracrine signaling within the brain, cells would have to rely on the steady diffusion of metabolites through the brain interstitial fluid. Using this transwell BBB model we previously have provided evidence in support of a model that direct communication between BMVEC and their neighboring astrocytes is required for proper regulation and transport of iron into the brain [7]. Here, we have extended these findings to understand the role BMVEC play in self-regulating their own iron transport through manipulation of a neighboring glial cell type.

**Figure 6:**
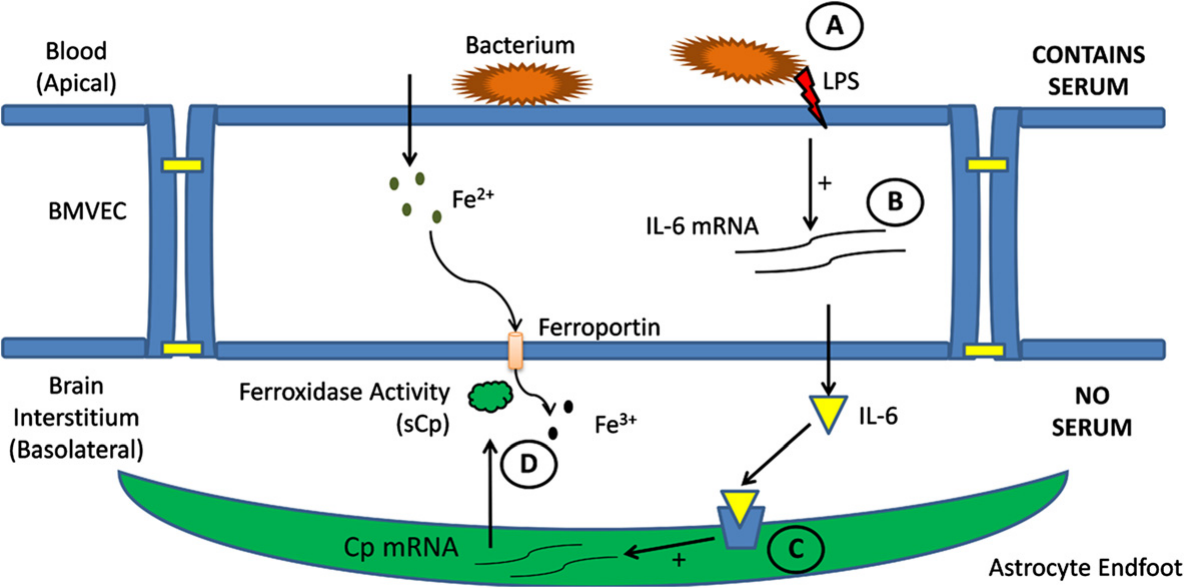
**Model of BMVEC-induced sequestration of iron from the blood to the brain interstitium under stimuli from bacterial endotoxin. (A)** The bacterial endotoxin Lipopolysaccharide (LPS) interacts with the apical surface of BMVEC resulting in enhanced gene expression of IL-6 **(B)**. **(C)** IL-6 secreted from the basolateral surface of BMVEC binds with its receptor on the cell surface of neighboring astrocytes triggering a signaling cascade leading to increased sCp gene expression. **(D)** This increase in sCp transcript relates to an increase in sCp protein secreted from astrocytes resulting in increased iron efflux from the basolateral surface of BMVEC.

In this model system regulation of C6 cell sCp expression is modulated by BMVEC-secreted IL-6 and IL-1β (Figure 3). This modulation is enhanced further when the apical (blood-side) of the brain capillary is exposed to bacterial endotoxin (LPS) (Figures 5 and 6A). Acute exposure to LPS induces increased gene expression of the cytokines IL-1β and IL-6 by hBMVEC (Figure 6B). The eventual increase in hBMVEC IL-6, but apparently not IL-1β, secretion into the interstitial space between hBMVEC and C6 cells yields activation of IL-6R endogenous to the glial cells. Transduction of this signal leads to downstream activation of Cp gene expression (Figure 6C). The soluble Cp splice variant gene product is secreted by the glial cells to act on Fpn endogenous to hBMVEC (Figure 6D). In theory, this model provides a mechanism by which inflammation may negatively affect BBB integrity via oxidative stress to the BMVEC caused by excessive extracellular iron in the interstitial space at the BBB. Previous data from the literature indicates that chronic inflammation may lead to a loss of BBB integrity [29]. Pathogenic organisms such as Streptococcus pneumonia (pneumococcal meningitis) may induce or take advantage of encephalitis and the breakdown of the BBB as a mechanism of entering the brain and CNS [30]. Thus, our data suggest that inflammation and/or pathogens in the brain cause hBMVEC to modulate their own basolateral iron efflux by supporting a cytokine-mediated activation of sCp expression in neighboring astrocytes.

While the mechanism of iron efflux from hBMVEC during inflammation described in Figure 6 is probable, a number of alternative mechanisms could be playing a role. For example, increased iron uptake by hBMVEC may lead to an IRE-IRP mediated increase in Fpn expression and iron efflux. Alternatively, inflammation may enhance the generation of nitric oxide leading to an increase in the expression of hBMVEC Fpn [31]. These hypotheses might be incorporated into future studies regarding hBMVEC response to inflammatory signals as it pertains to iron metabolism.

The model described in Figure 6 may also have implications in progressive neurodegeneration with aging. There is evidence that age correlates with increased brain expression of IL-1 protein and transcript [32]. Amyloid-β precursor protein (APP) present in both astrocytes and endothelial cells [33], may contribute to the Alzheimer’s disease pathology, and is positively regulated, in part, by IL-1 [34]. Also, data suggest that iron plays a role in the disease progression associated with Aβ plaque formation [35]. Reciprocally, a fragment of APP (FTP, for ferroportin-targeting peptide) stabilizes Fpn in the membrane of hBMVEC allowing for activation of brain iron import by exocytoplasmic ferroxidases [36]. In as much as we’ve now demonstrated that IL-1β and IL-6 increase sCp production in the brain, this sCp could act in concert with sAPP to increase the brain interstitial iron pool, thus exacerbating the progression of Aβ plaque formation. Clearly, further *in vivo* investigation into the role of IL-1β-mediated brain iron uptake is required before a link to the progression of Alzheimer’s disease can be made.

In summary, we have provided several lines of evidence demonstrating hBMVEC communicate with C6 glioma cells so as to provide the necessary exocrine factor, sCp, to hBMVEC in support of Fpn-dependent iron efflux and thus to brain iron accumulation. Our data indicate also that this cell signaling pathway appears to link system infection to brain iron metabolism in the context of our model BBB system.

### Experimental procedures

#### Cell culture and reagents

hBMVEC and C6 glioma cells were cultured in RPMI 1640 with 10% FBS and 10% NuSerum as previously described [37]. Experiments were performed in ThinCert tissue culture inserts (Transwells) (Greiner bio-one) or in 24-well tissue culture dishes as previously described [7]. Serum-free media was RPMI-1640 with the addition of sodium selenite and insulin as described previously [7]; no cytotoxicity or loss of adherence was noted in cells incubated with serum-free media for prolonged periods [7]. IL-1β, IL-6, and IRAP were purchased from Peprotech (Rocky Hill, NJ), SB203580 (Santa Cruz Biotechnology), SC144 and lipopolysaccharide (LPS) were purchased from Sigma-Aldrich.

### RT-PCR and qPCR

Total RNA was extracted from hBMVEC or C6 glioma cells using the TRIzol reagent (Invitrogen, Carlsbad, CA) as per the manufacturer’s instructions. RNA was purified and DNAse treated using the Direct-zol RNA Miniprep kit (Zymo-research) as per the manufacturer’s instructions. Pure RNA was then reverse-transcribed using SuperScript III Reverse Transcriptase (Invitrogen) along with gene-specific primers, and PCR was performed using the Qiagen OneStep RT-PCR kit. Quantitative PCR was performed as previously described [7] using SsoAdvanced SYBR Green Supermix (Bio-rad, Hercules, CA) and the Bio-rad CFX-96 real-time PCR instrument (Bio-Rad). In all cases β-actin was used as an internal control. Endpoint qPCR reactions were separated on a 1.7% agarose gel to confirm product size. Primers used for RT-PCR and qPCR are listed in Additional file 1: Table S1. Where applicable, 24 h incubations were used in an effort to maintain consistency with previous work utilizing this *in vitro* model BBB system [7].

### Immunoblots

Immunoblots were performed as previously described with minor changes [37]. Briefly, hBMVEC-conditioned basolateral media was concentrated and SDS-PAGE (12%) was used to separate the concentrate for the examination of IL-1β or IL-6. A 10% SDS-PAGE was used to separate proteins from C6-conditioned media for the examination of sCp. The membrane was blocked, and incubated with rabbit anti-IL-1β (ab2105) (Abcam, Inc., Cambridge, MA), rabbit anti-IL-6 (ab6672) (Abcam), or goat anti-Cp (Bethyl Laboratories, Inc.) overnight at 4°C and then incubated with secondary anti-rabbit or anti-goat HRP antibody (Santa Cruz Biotechnology). Blots were adjusted for brightness and contrast using Adobe Photoshop software (version 7.0).

### ^59^Fe efflux assay

Assays were performed with hBMVEC grown in 24-well tissue culture dishes as previously described [7,26]. Briefly, hBMVEC were loaded for 24 h with ^59^Fe in the presence of citrate (250 μM) and ascorbate (5 mM). After loading, cells were washed and incubated for an additional 24 h with fresh RPMI 1640 plus the appropriate reagents as indicated in the text and figure legends. Post-efflux, cells were washed twice with quench buffer and were lysed for analysis of ^59^Fe (CPM) and protein content. Cell-associated ^59^Fe values (LKB Wallac CompuGamma) were normalized by protein concentration. Protein concentrations were determined using the Pierce BCA protein assay (Thermo Scientific, Pittsburgh, PA) as per the manufacturer’s instructions.

### Statistical analysis

One-way ANOVA or paired t-test statistical analyses were used as indicated. Statistical analyses were performed using Prism Graphpad software version 5.0 (GraphPad Software, San Diego, CA).

## Additional file

Additional file 1: Figure S1. Apical chamber serum has no effect on C6 glioma sCp transcript abundance. **Figure S2.** hBMVEC sCp transcript abundance is positively regulates by C6 glioma cells. **Figure S3.** Modulation of C6 glioma sCp transcript abundance by LPS requires hBMVEC. **Table S1.** Primer list used for qPCR.

## Abbreviations

BBB: Blood–brain barrier
BMVEC: Brain microvascular endothelial cells
sCp: Soluble ceruloplasmin
Fpn: Ferroportin
IL-1β: Interleukin-1β
IL-6: Interleukin-6
hBMVEC: Human brain microvascular endothelial cells
IL-1R1: Interleukin-1 receptor 1
MAPK: Mitogen-activated protein kinase
IL-6R: interleukin-6 receptor
GPI-Cp: Glycosylphosphatidylinositol-linked ceruloplasmin
ECM: hBMVEC-conditioned media
IRAP: Interleukin-1 receptor antagonist protein
LPS: Lipopolysaccharide
APP: Amyloid-β precursor protein
FTP: Ferroportin-targeting peptide
CPM: Counts per minute

## References

[1] Salvador GA (2010). Iron in neuronal function and dysfunction. BioFactors.

[2] Levi S, Rovida E (2009). The role of iron in mitochondrial function. Biochim Biophys Acta.

[3] Stankiewicz JM, Brass SD (2009). Role of iron in neurotoxicity: a cause for concern in the elderly?. Curr Opin Clin Nutr Metab Care.

[4] Madsen E, Gitlin JD (2007). Copper and iron disorders of the brain. Ann Rev Neurosci.

[5] Altamura S, Muckenthaler MU (2009). Iron toxicity in diseases of aging: Alzheimer's disease, Parkinson's disease and atherosclerosis. J Alzheimers Dis.

[6] Rouault TA (2013). Iron metabolism in the CNS: implications for neurodegenerative diseases. Nat Rev Neurosci.

[7] McCarthy RC, Kosman DJ (2014). Glial cell ceruloplasmin and hepcidin differentially regulate iron efflux from brain microvascular endothelial cells. PLoS One.

[8] Nemeth E, Tuttle MS, Powelson J, Vaughn MB, Donovan A, Ward DM, Ganz T, Kaplan J (2004). Hepcidin regulates cellular iron efflux by binding to ferroportin and inducing its internalization. Science.

[9] De Domenico I, Ward DM, Langelier C, Vaughn MB, Nemeth E, Sundquist WI, Ganz T, Musci G, Kaplan J (2007). The molecular mechanism of hepcidin-mediated ferroportin down-regulation. Mol Biol Cell.

[10] Greco TM, Seeholzer SH, Mak A, Spruce L, Ischiropoulos H (2010). Quantitative mass spectrometry-based proteomics reveals the dynamic range of primary mouse astrocyte protein secretion. J Proteome Res.

[11] Persichini T, Maio N, di Patti MCB, Rizzo G, Colasanti M, Musci G (2010). Interleukin-1β induces ceruloplasmin and ferroportin-1 gene expression via MAP kinases and C/EBPβ, AP-1, and NF-κB activation. Neurosci Lett.

[12] di Patti MCB, Persichini T, Mazzone V, Polticelli F, Colasanti M, Musci G (2004). Interleukin-1β up-regulates iron efflux in rat C6 glioma cells through modulation of ceruloplasmin and ferroportin-1 synthesis. Neurosci Lett.

[13] Stylianou E, Saklatvala J (1998). Interleukin-1. Int J Biochem Cell Biol.

[14] Chang JW, Young DA, Coleman PD, O'Banion MK (2001). Two-dimensional gel analysis of secreted proteins induced by interleukin-1β in rat astrocytes. Neurochem Int.

[15] Corsini E, Dufour A, Ciusani E, Gelati M, Frigerio S, Gritti A, Cajola L, Mancardi GL, Massa G, Salmaggi A (1996). Human brain endothelial cells and astrocytes produce IL-1β but not IL-10. Scand J Immunol.

[16] Frigerio S, Gelati M, Ciusani E, Corsini E, Dufour A, Massa G, Salmaggi A (1998). Immunocompetence of human microvascular brain endothelial cells: cytokine regulation of IL-1ß, MCP-1, IL-10, sICAM-1 and sVCAM-1. J Neurol.

[17] Wilson HL, Varcoe RW, Stokes L, Holland KL, Francis SE, Dower SK, Surprenant A, Crossman DC (2007). P2X receptor characterization and IL-1/IL-1Ra release from human endothelial cells. Br J Pharmacol.

[18] Sidhu A, Miller PJ, Hollenbach AD (2011). FOXO1 stimulates ceruloplasmin promoter activity in human hepatoma cells treated with IL-6. Biochem Biophys Res Commun.

[19] Conley L, Geurs TL, Levin LA (2005). Transcriptional regulation of ceruloplasmin by an IL-6 response element pathway. Brain Res Mol Brain Res.

[20] Xu S, Grande F, Garofalo A, Neamati N (2013). Discovery of a novel orally active small-molecule gp130 inhibitor for the treatment of ovarian cancer. Mol Cancer Ther.

[21] Podor TJ, Jirik FR, Loskutoff DJ, Carson DA, Lotz M (1989). Human endothelial cells produce IL-6. Ann N Y Acad Sci.

[22] Sironi M, Breviario F, Proserpio P, Biondi A, Vecchi A, Van Damme J, Dejana E, Mantovani A (1989). IL-1 stimulates IL-6 production in endothelial cells. J Immunol.

[23] Carter DB, Deibel MR, Dunn CJ, Tomich CS, Laborde AL, Slightom JL, Berger AE, Bienkowski MJ, Sun FF, McEwan RN, Harris PKW, Yem AW, Waszak GA, Chosay JG, Sieu LC, Hardee MM, Zurcher-Neely HA, Reardon IM, Heinrikson RL, Truesdell SE, Shelly JA, Eessalu TE, Taylor BM, Tracey DE (1990). Purification, cloning, expression and biological characterization of an interleukin-1 receptor antagonist protein. Nature.

[24] Pita I, Jelaso AM, Ide CF (1999). IL-1β increases intracellular calcium through an IL-1 type 1 receptor mediated mechanism in C6 astrocytic cells. Int J Dev Neurosci.

[25] Cuenda A, Rouse J, Doza YN, Meier R, Cohen P, Gallagher TF, Young PR, Lee JC (1995). SB 203580 is a specific inhibitor of a MAP kinase homologue which is stimulated by cellular stresses and interleukin-1. FEBS Lett.

[26] McCarthy RC, Kosman DJ (2013). Ferroportin and exocytoplasmic ferroxidase activity are required for brain microvascular endothelial cell iron efflux. J Biol Chem.

[27] Raetz CRH, Whitfield C (2002). Lipopolysaccharide endotoxins. Annu Rev Biochem.

[28] Verma S, Nakaoke R, Dohgu S, Banks WA (2006). Release of cytokines by brain endothelial cells: A polarized response to lipopolysaccharide. Brain Behav Immun.

[29] Brooks TA1, Hawkins BT, Huber JD, Egleton RD, Davis TP (2005). Chronic inflammatory pain leads to increased blood–brain barrier permeability and tight junction protein alterations. Am J Physiol Heart Circ Physiol.

[30] Barichello T, Pereira JS, Savi GD, Generoso JS, Cipriano AL, Silvestre C, Petronilho F, Dal-Pizzol F, Vilela MC, Teixeira AL (2011). A kinetic study of the cytokine/chemokines levels and disruption of blood–brain barrier in infant rats after pneumococcal meningitis. J Neuroimmunol.

[31] Nairz M, Schleicher U, Schroll A, Sonnweber T, Theurl I, Ludwiczek S, Talasz H, Brandacher G, Moser PL, Muckenthaler MU, Fang FC, Bogdan C, Weiss G (2013). Nitric oxide–mediated regulation of ferroportin-1 controls macrophage iron homeostasis and immune function in Salmonella infection. J Exp Med.

[32] Sheng J, Mrak R, Griffin W (1998). Enlarged and phagocytic, but not primed, interleukin-1 alpha-immunoreactive microglia increase with age in normal human brain. Acta Neuropathol.

[33] Siman R, Card JP, Nelson RB, Davis LG (1989). Expression of beta-amyloid precursor protein in reactive astrocytes following neuronal damage. Neuron.

[34] Goldgaber D, Harris HW, Hla T, Maciag T, Donnelly RJ, Jacobsen JS, Vitek MP, Gajdusek DC (1989). Interleukin 1 regulates synthesis of amyloid beta-protein precursor mRNA in human endothelial cells. Proc Natl Acad Sci U S A.

[35] Bolognin S, Messori L, Drago D, Gabbiani C, Cendron L, Zatta P (2011). Aluminum, copper, iron and zinc differentially alter amyloid-Aβ1–42 aggregation and toxicity. Int J Biochem Cell Biol.

[36] McCarthy RC, Park YH, Kosman DJ (2014). sAPP modulates iron efflux from brain microvascular endothelial cells by stabilizing the ferrous iron exporter ferroportin. EMBO Rep.

[37] McCarthy RC, Kosman DJ (2012). Mechanistic analysis of iron accumulation by endothelial cells of the BBB. Biometals.

