# Supplementary material for: Activation of C6 glioblastoma cell ceruloplasmin expression by neighboring human brain endothelia-derived interleukins in an in vitro blood–brain barrier model system
Source: Cell Commun Signal. 2014 Oct 14;12:65. doi: 10.1186/s12964-014-0065-7 (PMC4200123; doi:10.1186/s12964-014-0065-7)
Supplement: Additional file 1: Figure S1. — Apical chamber serum has no effect on C6 glioma sCp transcript abundance. Figure S2. hBMVEC sCp transcript abundance is positively regulates by C6 glioma cells. Figure S3. Modulation of C6 glioma sCp transcript abundance by LPS requires hBMVEC. Table S1. Primer list used for qPCR. [file 12964_2014_65_MOESM1_ESM.pdf]

Additional Material

Figure Legends

Figure S1. Apical chamber serum has no effect on C6 glioma sCp transcript abundance. qPCR of sCp transcript from total RNA collected from C6 glioma cells grown alone in serum-free media (C6) or grown alone in transwell with serum-free media in the basal chamber and media plus serum in the apical chamber (/-/C6). A paired t-test was used to determine the data are not significantly different. Data are represented as means ± s.d. (n = 3, technical replicates).

Figure S2. hBMVEC sCp transcript abundance is positively regulates by C6 glioma cells. qPCR of hBMVEC sCp transcript from total RNA collected from hBMVEC grown either alone (EC) or distal to C6 glioma cells (EC/-/C6). A paired t-test was used to determine the significance of the data. *P < 0.05. Data are represented as means ± s.d. (n = 3, technical replicates).

Figure S3. Modulation of C6 glioma sCp transcript abundance by LPS requires hBMVEC. Total RNA was isolated from C6 glioma cells seeded alone in transwell with or without the addition of LPS (100 µg/mL) to the apical chamber for 24h. Soluble Cp transcript abundance was assessed via qPCR. One-way ANOVA statistical analysis was used to determine the significance of the data. Data are represented as means ± s.d. (n = 3, technical replicates).

Figure S1

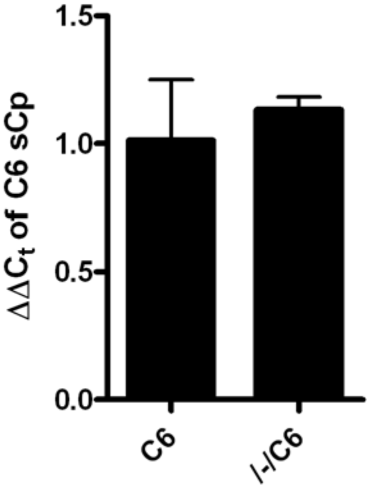

Figure S2

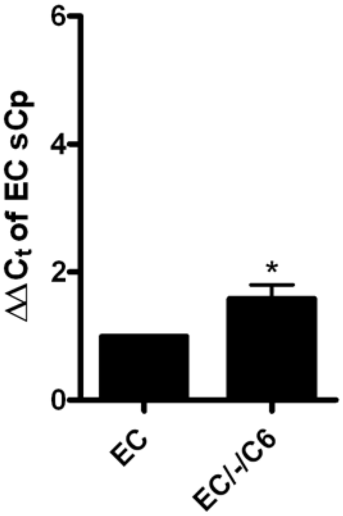

Figure S3

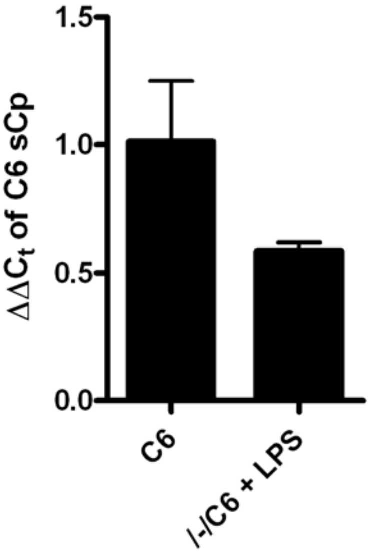

Table S1. Primer list used for qPCR

Transcript	Forward Primer	Reverse Primer
β-actin	GGGTCAGAAGGACTCCTACG	GGTCTCAAACATGATCTGGG
Rat sCp	TCCACTGCCATGTGACTGAC	TCGGCATTACCAATTCCCTCA
Rat GPI-Cp	GGTTACTCCACTGCCATGTG	GTGTATAGAGTATGTTCCAGG
Isoform non-specific Cp	TGGTTTATCGTGAGTACACAG	TCTGCCCAAATGACAGGACC
Human IL-1β	TCACTTAAAGCCCGCCTGAC	GGAAGCGGTTGCTCATCAGA
Human IL-6	CCACCGGGAACGAAAGAGAA	GAGAAGGCAACTGGACCGAA
Human sCp	TCCCTGGAACATACCAAACC	CCAATTTATTTCATTCAGCCGA

Except where indicated primer pairs are species non-specific (rat/human)
